# Early Development of Cardiac Fibrosis in Young Old-Father Offspring

**DOI:** 10.1155/2022/8770136

**Published:** 2022-09-19

**Authors:** A. Ismail, Y. Saliba, N. Fares

**Affiliations:** Physiology and Pathophysiology Research Laboratory, Pole of Technology and Health, Faculty of Medicine, Saint Joseph University, Beirut, Lebanon

## Abstract

Cardiac aging is characterized by progressive fibrosis. Epidemiological studies have found that advanced paternal age is associated with an increased risk of heart failure in the next generation. This study is aimed at evaluating the effect of paternal age, in the young male rat progeny, on cardiac phenotype under circulatory stress conditions. Offspring rats were obtained by mating old males (24 months old) with young females (two months old) and by mating young males (two months old) with the same young females. Hypertension was induced in old father offspring (OFO) rats and young old father (YFO) offspring rats using L-NAME (N(*ω*)-nitro-L-arginine methyl ester). The OFO L-NAME rats showed a high blood pressure phenotype associated with substantial cardiac hypertrophy and an exacerbation of cardiac fibrosis compared to the YFO L-NAME rats. Histological analysis of heart tissue showed an expansion of the extracellular matrix, with fibroblasts displaying markers of epicardial origin (Tcf21, Tbx18, and Wt1) in the OFO group. Moreover, western blot and protein phosphorylation antibody array identified the TGF-*β*2 receptor pathway as preferentially activated in aged hearts as well as in OFO cardiac tissue treated with L-NAME. In addition, old father offspring rats (OFO+OFO L-NAME) had increased cardiac DNA methylation. In young hypertensive progeny, advanced paternal age at conception may be a risk factor for early progression towards cardiac fibrosis. An intergenerational transmission may be behind the paternal age-related cardiac remodeling in the young offspring.

## 1. Introduction

Physiological aging is associated with organ remodeling and fibrosis [1], leading to progressive functional decline in various organs [2], i.e., the heart, lungs, liver, kidneys, and musculoskeletal muscle. Cardiac fibrosis is defined as the excessive production and deposition of extracellular matrix proteins, mainly collagen, in the interstitium [3]. TGF-*β* is a profibrotic signaling pathway regulating the activation of fibroblasts, the main cellular effectors in this process [4]. Moreover, TGF-*β* has been found to regulate mesenchymal cell proliferation and migration into target tissue during fibrosis [5].

Aging is a nonmodifiable cardiovascular risk [6], and many cardiovascular diseases progress to heart failure and lead to the development of cardiac fibrosis [7]. Epigenetics play a central role in aging-related cardiac fibrosis [8] by modulating fibroblast differentiation and proliferation [9] and regulating signaling pathways [10]. Moreover, mounting evidence has demonstrated that these epigenetic modifications, transmitted by sperm [11] and through an intergenerational effect [12], are increasing the susceptibility to aging-related disorders in the offspring. Advanced paternal age observed in mice has been associated with reduced lifespan and the development of aging traits in offspring at a younger age [13]. Moreover, several epidemiological studies have demonstrated that advanced paternal age is associated with an increased risk of developing cardiovascular diseases and heart failure in the offspring [14–16]. However, the influence of advanced paternal age on cardiac fibrogenesis in the offspring remains unclear. Therefore, this study is aimed at evaluating the effect of paternal age, in the young male rat progeny, on the cardiac phenotype under circulatory stress conditions.

## 2. Materials and Methods

### 2.1. Ethical Approval

The present work was approved by the Ethical Committee of the Saint Joseph University of Beirut, Lebanon. Protocols were carried out according to the “Guiding Principles in the Care and Use of Animals” approved by the Council of the American Physiological Society and in adherence to the “Guide for the Care and Use of Laboratory Animals” published by the US National Institutes of Health (NIH Publication. N. 85-23, revised 1996) and by the European Parliament Directive 2010/63 EU.

### 2.2. Animals and Study Groups

The study was conducted using albino outbred Wistar rats, aged four and 24 months, divided into six groups of five animals each, as previously described [13]: (i) old father (OF) and (ii) young father (YF). Old father offspring was obtained from mating OF males with young females. This group was divided into (iii) old father offspring (OFO) and (iv) old father offspring treated with L-NAME (OFO L-NAME) (N(*ω*)-nitro-L-arginine methyl ester) (Sigma-Aldrich, St Louis, Missouri, USA), at a dose of 50 mg.kg^−1^.day^−1^ per rat for 45 days until sacrifice. L-NAME was administered orally in the drinking water and renewed daily. Young father offspring was obtained from mating YF rats with the same young females previously mated with OF rats, after a two-month latency period. This group was divided into (v) young father offspring (YFO) and (vi) young father offspring L-NAME (YFO L-NAME). This hypertension animal model with systemic endothelial nitric oxide inhibition is well-established for inducing and enhancing cardiac fibrosis independently of changes in peripheral blood pressure and LV pressure [17, 18]. Males interacted with females exclusively during mating and never with their litters. Rat offspring were left alone with their dam until they were weaned at 21 days of age. Animals were housed in a controlled environment, with stable temperature (24 ± 2°C) and humidity (45-55%) conditions, and were exposed to a 12 : 12 h light-dark cycle.

### 2.3. Blood Pressure Measurement

Systolic blood pressure was measured using the noninvasive tail-cuff method (IITC, CA, USA). Briefly, awake rats were placed in acrylic cylinders in a heated chamber at 29°C for ten minutes before acquiring the data. All measurements were taken before sacrifice, in triplicate for each condition, between 10 a.m. and 12 p.m., to avoid variations in blood pressure due to the circadian cycle.

### 2.4. Transthoracic Echocardiography

Transthoracic echocardiography was performed using the SonoScape S2V high-resolution color Doppler ultrasound system equipped with a 10 MHz L741 probe (SonoScape Co., Shenzhen, China) adapted for mice and rats. Just before sacrifice, rats were anesthetized with isoflurane (5% for induction and 3% for maintenance) (Abbott Laboratories, Chicago, IL, USA) at a flow of 1 L.min^−1^ using an EZ-SA800 Anesthesia Single Animal System (E-Z Systems, Pennsylvania, USA). Left ventricular (LV) parasternal long-axis 2D view in M-mode was performed at the papillary muscle to assess LV wall thicknesses and internal diameters. From this view, the LV posterior wall end diastole (LVPWd), the interventricular septal end diastole (IVSd), the LV internal diameter end-diastole and end-systole (LVIDd and LVIDs), the end-diastolic volume (EDV), the end-systolic volume (ESV), and the ejection time (ET) were obtained. The ejection fraction (EF %) and fractional shortening (FS) were calculated by the Teichholz method. Measurements were obtained by two independent operators blinded to the conditions.

### 2.5. Animal Sacrifice and Histological Analysis

Rats were sedated by intraperitoneal injection using a mixture of ketamine 75 mg.kg^−1^ (Interchemie, Waalre, Holland) and xylazine 10 mg.kg^−1^ (RotexMedica, Trittau, Germany). The depth of anesthesia was verified by the pedal withdrawal reflex. When animals were completely nonresponsive to toe pinching, their hearts were removed. Each heart was cut into half through a midsagittal plane. One-half was kept at -80°C for protein and DNA extraction, whereas the other half was cut through a sagittal plane with one part kept in 10% neutral formalin solution and the other part embedded in optimal cutting temperature (OCT) compound.

The formalin-fixed tissue was embedded in paraffin, and sections of 4 *μ*m thickness were cut and then stained with hematoxylin-eosin (HE) and Masson's trichrome (Sigma-Aldrich, St. Louis, MO, USA). Representative pictures were taken using a VanGuard High-Definition Digital Camera (VEE GEE Scientific, IL, USA).

Cryosections of 4 *μ*m thickness were cut from the OCT-embedded hearts. Wheat germ agglutinin staining (594 nm) was done according to the manufacturer's protocol (ThermoFisher Scientific, Waltham, MA, USA). Primary antibodies were as follows: *α*-SMA (ab32575; 1/100; Abcam, Cambridge, UK), Tcf21 (NBP1-88637; Novus Biologicals, Littleton, CO, USA), and PDGFR*α* (sc-398206; Santa Cruz Biotechnology, Dallas, TX, USA). The slides were then incubated with the preadsorbed secondary antibody (goat anti-rabbit and anti-mouse IgG H&L Alexa Fluor 594), mounted with fluoroshield mounting medium (Abcam, Cambridge, UK), and pictures were taken using an Axioskop 2 immunofluorescence microscope (Carl Zeiss Microscopy GmbH, Jena, Germany) equipped with a CoolCube 1 CCD camera (MetaSystems, Newton, Massachusetts, USA). All image analysis and quantifications were done using ImageJ.

### 2.6. Assessment of Global Cardiac DNA Methylation and Hydroxymethylation

Blood samples were collected, at the time of sacrifice, in EDTA tubes. Plasma was obtained by centrifugation at 4,500 rpm for 10 min. Genomic DNA was extracted from hearts using the TRIzol method, and DNA quality was checked by agarose gel electrophoresis and SYBR Safe DNA stain (ThermoFisher Scientific, Waltham, MA, USA). Global cardiac methylation and hydroxymethylation (ab117128 and ab117130, Abcam, Cambridge, UK) were measured by the ELISA technique according to the manufacturer's protocols. Genomic DNA was bound to the plate wells before the specific primary antibodies were added.

### 2.7. Antibody Array for Protein Expression

The TGF-*β* protein phosphorylation antibody array was from Full Moon Biosystems and consisted of an ELISA-based antibody array platform comprising 174 highly specific antibodies targeting proteins involved in the TGF-*β* signaling pathway (Full Moon Biosystems, Sunnyvale, California, USA). The protocol was carried out exactly as instructed by the manufacturer. In summary, the array entails four key steps: (i) protein extraction using nondenaturing lysis buffer, (ii) biotinylation of protein samples, (iii) incubation of labeled samples with antibody array, and (iv) detection by Cy3-conjugated streptavidin. Cardiac tissue extracts were prepared using Complete Lysis-M EDTA-Free Lysis buffer (Roche, South San Francisco, CA, USA) with phosphatase inhibitor cocktail (*β*-glycerophosphate, sodium orthovanadate, sodium fluoride, and sodium pyrophosphate; Sigma-Aldrich). The images were acquired by the manufacturer. Signal intensity was determined by Full Moon Biosystems on a GenePix 4000B Imager (Molecular Devices, San Jose, California, USA) using GenePix Pro software (Molecular Devices, San Jose, California, USA) by an objective scorer blinded to sample identity. For each antibody, the average signal intensity of replicate spots was normalized to the median value of average signal intensity for all antibodies on the array. The fold change represented the ratio of distinct groups based on normalized data. GAPDH and beta-actin were used as positive controls and bovine serum albumin as a negative control.

### 2.8. Protein-Protein Interaction Network Analysis

The STRING (https://string-db.org) database, which includes both known and anticipated protein-protein interactions, was used to predict physical and functional interactions between proteins [19]. The protein-protein interaction networks were built using active interaction sources such as text mining, experiments, databases, and coexpression, as well as a species restriction of “Rattus norvegicus” and an interaction score > 0.4. To display the protein-protein interaction network, Cytoscape 3.9.0 software with the ClueGO v.2.5.8 plugin was utilized. To identify highly linked parts of the network, the following criteria were used: minimum size = 5, minimum density = 0.05, and edge weights = combined score. If missing edges are expected to weigh zero, the minimum density indicates the average edge weight inside the cluster, and edge weights define the weight of each edge. The proteins are represented by the nodes, while the interactions are represented by the edges.

### 2.9. Western Blotting

Protein samples (20 *μ*g) were separated in a vertical electrophoresis system and transferred to polyvinylidene fluoride (PVDF) membranes (Bio-Rad Laboratories Inc., Irvine, CA, USA). Membranes were blocked with 5% BSA. The blots were then incubated with the primary antibodies: NFATc3 (sc-8321) and phospho-Ser168/170 NFATc4 (sc-32630) (Santa Cruz Biotechnology, Dallas, TX, USA); phospho-Ser165 NFATc3 (p-NFATc3) (ab59204), NFATc4 (ab183117), SMAD2 (ab63576), phospho-S467 SMAD2 (p-SMAD2) (ab53100), SMAD3 (ab28379), and phospho-S423/S425 SMAD3 (p-SMAD3) (ab52903) (Abcam, Cambridge, UK); and ERK1/2 (#4695), phospho-Thr202/Tyr204 ERK1/2 (#4370), p38 (#8690), phosphoThr180/Tyr182 p38 (#4511), and glyceraldehyde-3-phosphate dehydrogenase (GAPDH) (#5174) (Cell Signaling Technology, Danvers, Massachusetts, USA). Following anti-rabbit secondary antibody incubation, membranes were visualized using ECL (Bio-Rad Laboratories, Inc., Irvine, CA, USA), and signals were detected by an imaging system equipped with a CCD camera (Omega Lum G, Aplegen, Gel Company, SF, USA). Signal intensities of bands in the immunoblots were quantified using Image Studio Lite Ver. 5.2 (LI-COR Biosciences, Lincoln, NE, USA) analysis software. Four western blots were performed for each protein and condition.

### 2.10. Statistical Analysis

Statistical analysis was conducted using GraphPad Prism 9 and data was presented as mean ± SEM. The Shapiro-Wilk normality test was used to check whether populations followed a Gaussian distribution. When Gaussian distribution was met, ordinary one-way ANOVA was used for multiple comparisons of the means, followed by post hoc Tukey's test to determine which groups accounted for overall ANOVA significance. When data were skewed, the Kruskal-Wallis one-way ANOVA on ranks was used, followed by Dunn's multiple comparison tests. For representation of statistical significance, ∗, ∗∗, ∗∗∗, and ∗∗∗∗ indicate *p* values of <0.05, <0.01, <0.001, and <0.0001, respectively.

## 3. Results

### 3.1. Exacerbation of Left Ventricular Remodeling in Hypertensive Old Father Offspring Rats

**Figure 1:**
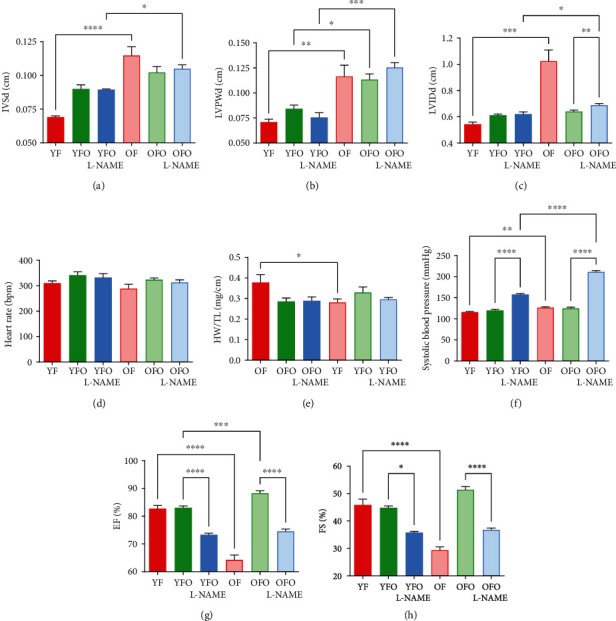
Cardiac hypertrophy in hypertensive old father offspring. Evaluation of heart and echocardiographic parameters. Histograms showing (a) IVSTd: end-diastolic interventricular septal thickness in centimeter (cm), (b) LVPWd: end-diastolic left ventricular posterior wall thickness in cm, (c) LVIDd: end-diastolic left ventricular internal diameter in cm, (d) HR: heart rate in beats per minute (bpm), (e) HW: heart weight and TL: tibia length (mg/cm), (f) systolic blood pressure histogram in mm of mercury (mmHg), and (g, h) echocardiographic parameters for cardiac function evaluation in different animal groups. EF: ejection fraction; FS: fractional shortening, in %. ^∗^*p* < 0.05, ^∗∗^*p* < 0.01, ^∗∗∗^*p* < 0.001, and ^∗∗∗∗^*p* < 0.0001.

Cardiac hypertrophy was present in OFO rats and more pronounced in OFO L-NAME. Cardiac hypertrophy was eccentric with an increased interventricular septal diameter (Figure 1(a)), left ventricular posterior wall thickness (Figure 1(b)), and end-diastolic left ventricular internal diameter (Figure 1(c)). The echocardiographic parameters in OFO L-NAME rats showed a similar pattern to those measured in OF rats (Figures 1(a)–1(c)). However, the heart rate of OFO rats was comparable between groups (Figure 1(d)). In addition, similar results were observed for heart weight over tibia length ratio except for the OF group (Figure 1(e)). Measurement of systolic blood pressure revealed that OFO rats were more susceptible to L-NAME-induced hypertension in comparison to other groups including YFO L-NAME (Figure 1(f)). In addition, OFO and YFO rats treated with L-NAME had reduced EF and FS in comparison to OFO and YFO rats, respectively (75 ± 1% vs. 88 ± 1%, 73 ± 1% vs. 83 ± 1%, *p* = 0.0001), and were similar to OF rats (64 ± 2%) (Figures 1(g) and 1(h)).

### 3.2. Marked Cardiac Fibrosis in L-NAME-Treated Old Father Offspring Rats

**Figure 2:**
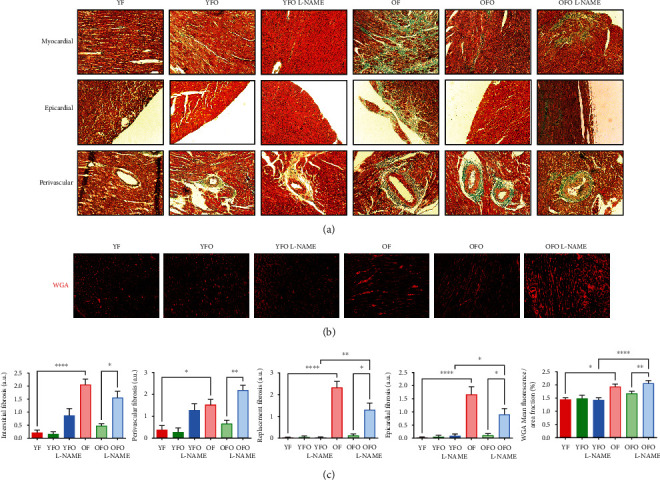
Cardiac remodeling in hypertensive old father offspring. (a) Perivascular coronary, epicardial, and myocardial histological sections stained with Masson's trichrome were obtained from different animal groups. The green stains represent the fibrotic areas. (b) Representative images of immunofluorescence staining (594 nm) for wheat germ agglutinin (WGA) in the hearts of different animal groups. (c) Quantifications of interstitial reactive, perivascular, replacement, and epicardial fibrosis, as well quantifications of immunofluorescence staining for WGA. Scale bars: 100 *μ*m in (a) and 50 *μ*m in (b). Magnifications: ×100 in (a) and ×200 in (b). ^∗^*p* < 0.05, ^∗∗^*p* < 0.01, ^∗∗∗^*p* < 0.001, and ^∗∗∗∗^*p* < 0.0001.

To investigate the location and extent of cardiac fibrosis, histological analysis of left ventricle sections stained with Masson's trichrome was conducted (Figure 2). Myocardial, epicardial, and perivascular regions were examined for total collagen deposition. Myocardial regions were evaluated for interstitial and replacement fibrosis, whereas perivascular fibrosis was defined as collagen accumulation in the adventitia of coronary arteries. Hearts from OFO L-NAME rats showed a fibrotic phenotype in comparison to OFO rats and displayed a similar fibrotic pattern to OF rats (Figures 2(a) and 2(c)). However, young rats and their offspring presented no signs of myocardial or epicardial fibrosis and had coronary vessels with thin adventitia (Figures 2(a) and 2(c)). Moreover, YFO L-NAME presented a nonsignificant increase in interstitial and perivascular fibrosis (Figures 2(a) and 2(c)). Finally, the expansion of the cardiac interstitium was evaluated using WGA staining. In concordance with the previous histological results, the left ventricle of OFO L-NAME had a marked interstitial expansion in comparison to OFO, with a similar pattern to the one seen in OF rats (Figures 2(b) and 2(c)).

### 3.3. Expansion of Resident Cardiac Fibroblasts from an Epicardial Origin in L-NAME-Treated Old Father Offspring

**Figure 3:**
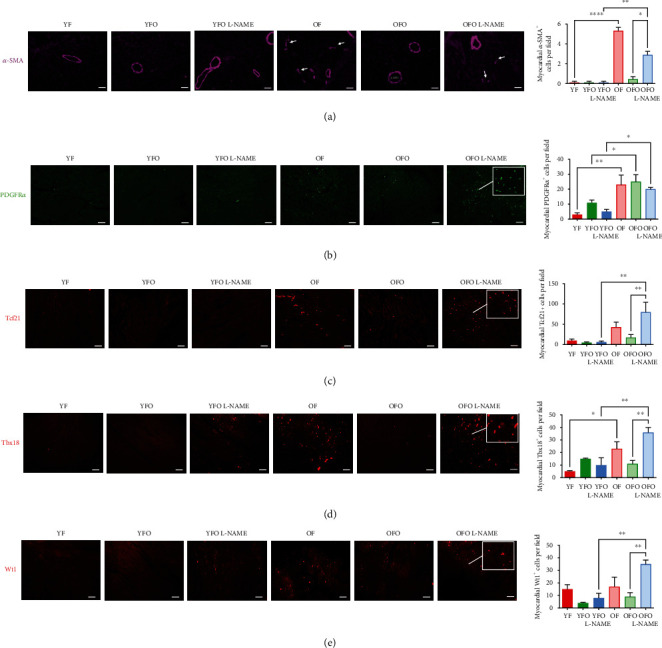
Cardiac remodeling in hypertensive old father offspring. (a) Immunofluorescence staining (594 nm) for *α*-SMA of histological sections obtained from the hearts of different animal groups, as well as quantifications of the respective stained zones. White asterisks indicate coronary vessels. (b–e) Immunofluorescence staining (488 nm) for PDGFR*α*, Tcf21, Tbx18, and Wt1 of histological sections obtained from the hearts of different animal groups, as well as quantifications of the respective stained zones. The insets in all of the panels represent a higher magnification to highlight the interstitial fibroblast distribution. Scale bars: 50 *μ*m. Magnifications: ×200. ^∗^*p* < 0.05, ^∗∗^*p* < 0.01, ^∗∗∗^*p* < 0.001, and ^∗∗∗∗^*p* < 0.0001.

The involvement of cardiac fibroblasts as major contributors to cardiac fibrosis was investigated by focusing on the lineage and distribution of these cells in the various rat groups (Figure 3). A significant increase in *α*-SMA expression was seen in OF and OFO L-NAME hearts. The distribution was patchy and predominantly concentrated in the replacement fibrosis zones. This myofibroblast marker, *α*-SMA, was exclusively present around coronary vessels in the remainder of the rat groups (Figure 3(a)). PDGFR*α*^+^-cell density remained relatively unchanged when comparing YF versus OF/OFO, and L-NAME did not affect the proliferation and expansion of these cells. When compared to the young groups, OF/OFO/OFO L-NAME had significantly higher PDGFR*α*^+^-cell densities (Figure 3(b)). Interstitial cells expressing epicardial progenitor transcription factors, Tcf21, Tbx18, and Wt1, were all present at relatively low densities in the YF, YFO, and YFO L-NAME hearts. OF hearts, on the other hand, presented an increase in both Tcf21 and Tbx18 markers, whereas L-NAME treatment raised the density of cells expressing all three epicardial markers in OFO rats only (Figures 3(c)–3(e)).

### 3.4. Activation of Cardiac TGF-*β* and Its Downstream ERK Protein Pathway in L-NAME-Treated Old Father Offspring

**Figure 4:**
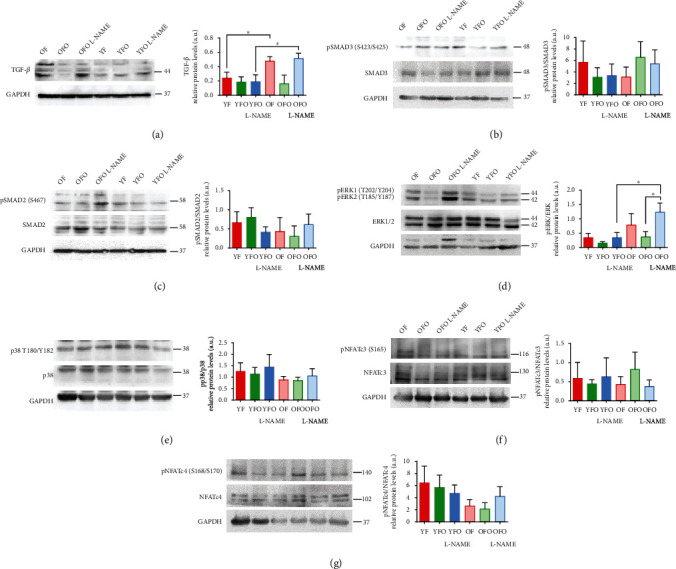
Upregulation of TGF-*β* and its downstream ERK protein in L-NAME old father offspring. (a–g) Western blots and quantifications of TGF-*β*1, SMAD3, p-SMAD3, SMAD2, p-SMAD2, ERK, pERK, NFATc3, pNFATc43, p38, pp38, NFTAc4, and pNFTAc4, respectively, in the hearts of the different animal groups, with GAPDH as an internal control (*n* = 3 for each condition). TGF-*β*1: transforming growth factor beta 1; SMAD2/3: mothers against decapentaplegic homologs 2/3; NFATC3/4: nuclear factor of activated T cells 3/4; ERK: extracellular signal-regulated kinase; p: phospho; GAPDH: glyceraldehyde-3-phosphate dehydrogenase; a.u.: arbitrary unit. All quantitative data are reported as mean ± SEM. ^∗^*p* < 0.05.

Proteins extracted from the hearts were used to study the activation of both canonical and noncanonical TGF-*β* with its downstream profibrotic signaling proteins, i.e., SMAD2/3, ERK1/2, p38, and NFATc3/c4 (Figure 4). Between the four offspring groups of interest, TGF-*β* expression was only increased in OFO L-NAME rats to a similar extent to that of OF (Figure 4(a)). The phosphorylated form of SMAD3 (p-SMAD3) at serine 423 and 425 and of SMAD-2 (p-SMAD2) at serine 467 were both increased in the OFO groups in comparison to YFO. When phosphorylated SMAD3 and SMAD2 were normalized to total SMAD3 and SMAD2, respectively (p-SMAD3/SMAD3 and pSMAD2/SMAD2), protein activation status was unchanged over all the groups (Figures 4(b) and 4(c)).

The unphosphorylated forms of ERK1/2 increased in OFO group and decreased in YFO. The phosphorylated form of ERK1/2 at T202/Y204 and T185/Y187 increased only in OFO L-NAME. When phosphorylated ERK1/2 was normalized to total ERK1/2, protein activation status was significantly higher in OFO L-NAME in comparison to YFO L-NAME and OFO, respectively (Figure 4(d)). The normalized ratio of phosphorylated total protein of NFATc3, p38, and NFATc4, respectively, showed a stable expression among the different groups (Figures 4(f) and 4(g)).

### 3.5. Cluster Activation of TFG-*β*2 Signaling in Hypertensive Old Father Offspring Rats

**Figure 5:**
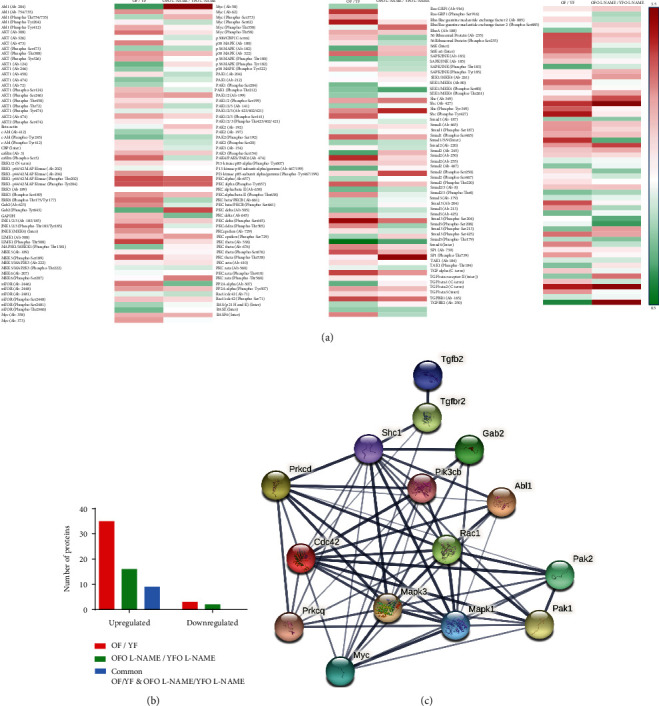
Identification of activated proteins in the TGF-*β* signaling pathway. (a) Heat maps of proteins identified in the TGF-*β* pathway. Differentially phosphorylated proteins were color-coded, with red representing higher levels of protein expression and green suggesting lower levels. (b) Histogram showing the number of upregulated and downregulated proteins in the TGF-*β* pathway in OF, YF, OFO L-NAME, and YFO L-NAME animal groups. (c) Analysis of protein-protein interaction networks within the TGF-*β* pathway. The STRING Protein-Protein Interaction Network (v.11.0) program creates networks that show interactions between proteins identified when comparing OFO L-NAME to YFO L-NAME, analogous to OF versus YF. The interaction's intensity is reflected in the line thickness.

A TGF-*β* phospho-protein array, consisting of 174 target proteins, was performed to explore the role of the TGF-*β* signaling pathway in cardiac fibrosis (Figure 5). Antibodies against phosphorylated kinases, adaptor proteins, and transcription factors, as well as antibodies against the total proteins of these molecules, are included in this phospho-protein array, which was used to seek additional molecules implicated in the TGF-*β* signaling cascade. OF rats were compared to YF and OFO L-NAME were compared to YFO L-NAME. TGF-*β*2 receptor and its ligand were upregulated in OFO L-NAME group, with a 3.5-fold increase in comparison to YFO L-NAME (Figure 5(a)). Overall, 35 proteins were upregulated in OF versus YF rats, 15 in OFO L-NAME versus YFO-LNAME, and nine proteins were commonly upregulated in the two comparative patterns. Interestingly, no common proteins were downregulated in OF versus YF and OFO L-NAME versus YFO L-NAME groups (Figure 5(b)).

TGFB2 was identified as a protein hub preferentially activating the isoform 2 of TGFBR in aged hearts as well as in the OFO cardiac tissue treated with L-NAME (Figure 5(c)). Downstream signaling included noncanonical TGFB pathways, such as the adaptor protein Shc1, the docking protein Gab2 mediating the interaction between the receptor and downstream phosphoinositide 3-kinase (Pik3cb), along with the Rac1/Cdc42-Pak1/2 pathway interacting with Pi3k3cb. On the other hand, protein kinases C (Prkcd/Prkcq) with their downstream effectors, Mapk1 (ERK2) and Mapk2 (ERK1), as well as their respective transcription factor Myc, were also promoted by TGFBR2. Finally, the tyrosine kinase Abl1 was also highly induced in OF and OFO L-NAME and exhibited substantial interactions with Shc and Rac1 proteins (Figure 5(c)).

### 3.6. Cardiac Methylation Profiles in Old Father Offspring

**Figure 6:**
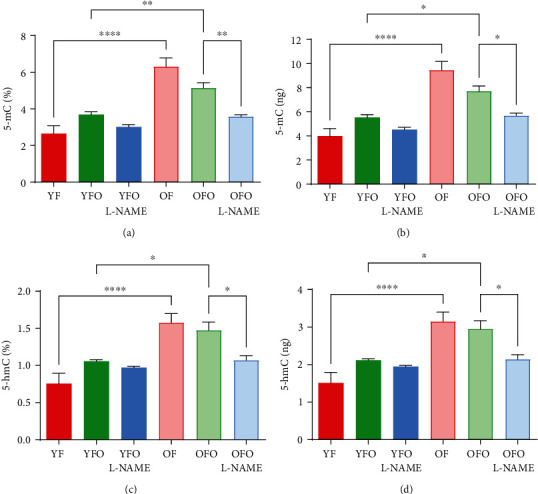
Increased cardiac DNA methylation in old father offspring. Cardiac 5-methylcytosine (5-mC) and 5-hydroxymethylcytosine (5-hmC) levels in different animal groups. Results are reported as mean ± SEM. ^∗^*p* < 0.05, ^∗∗^*p* < 0.01, ^∗∗∗^*p* < 0.001, and ^∗∗∗∗^*p* < 0.0001.

To explore the epigenetic modifications in the myocardium, methylation and hydroxymethylation measurements were conducted (Figures 6(a)–6(d)). Overall, the percentage and total DNA methylation and hydroxymethylation were the highest in OF rats (Figures 6(a)–6(d)). The OFO group showed significant increases in both methylation and hydroxymethylation rates in comparison with YFO and OFO L-NAME rats, respectively. Interestingly, the rate of methylation and hydroxymethylation was comparable between OFO L-NAME and YFO L-NAME (Figures 6(a)–6(d)).

## 4. Discussion

**Figure 7:**
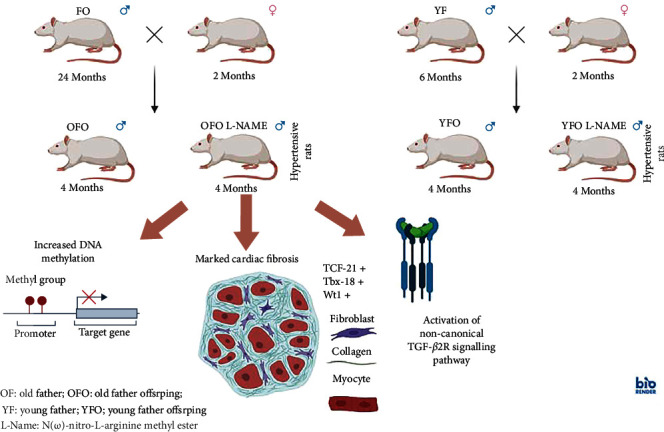
Effect of older paternal age at conception on the exacerbation of cardiac remodeling and development of myocardial fibrosis in the young rat progeny, through activation of the TGF-*β* signaling pathway, particularly in the case of chronic circulatory stress. The early development of cardiac remodeling associated with paternal age in the young progeny appears to be transmitted intergenerationally. Clinically, the early onset of heart failure in young hypertensive offspring may be correlated with advanced paternal age during conception.

Myocardial aging is characterized by left ventricular fibrosis leading to heart dysfunction. Epidemiological studies have found that advanced parental age was associated with an increased risk of heart failure in the next generation. Here, we demonstrate the influence of advanced paternal parental age, at conception, on the exacerbation of cardiac remodeling and development of myocardial fibrosis by activation of the TGF-*β* signaling pathway, especially in the event of chronic circulatory stress (Figure 7).

The echocardiographic parameters demonstrated the presence of marked cardiac hypertrophy in young OFO rats, with comparable results to OF rats despite the age difference. These significant changes appear to be related to the age of the father and not to L-NAME, which is used to induce high blood pressure without compensatory cardiac hypertrophy [20]. HW/TL was comparable between groups, despite the presence of echocardiographic signs of hypertrophy, which suggest the presence of cellular apoptosis [21] in the hearts of old father offspring rats. Additionally, advanced paternal age seemed to increase the susceptibility to hypertension in the offspring, with very high systolic pressures. These results suggest that advanced paternal age renders the offspring vulnerable to hypertension-induced left ventricular remodeling and a decline in cardiac function.

Aging-related myocardial fibrosis leads to an expansion of the extracellular matrix (ECM) [22]. Cardiac fibroblasts, the main contributors to cardiac fibrosis, are activated after injury (i.e., pressure overload and myocardial infarction) leading to an expansion of the ECM, mainly through excessive production and deposition of collagen fibers in the interstitium. Young OFO hearts showed a significant increase in all types of cardiac fibrosis with a comparable profile to OF rats and an expansion of the ECM, particularly after treatment with L-NAME. An increased afterload, which is an elevated of systolic blood pressure, will stimulate cardiac fibroblasts and increase collagen formation and cause remodeling of the myocardium with a disproportionate increase in fibrous tissue. Our results align with the published literature regarding the development of cardiac fibrosis in a hypertension animal model [23]. Nevertheless, we did not evaluate the correlation between the severity of hypertension and the extent of cardiac fibrosis in individual rats. Moreover, activated fibroblasts differentiate into myofibroblasts under stress or in heart failure [24]. OFO L-NAME rats demonstrated an increase in myofibroblasts expressing *α*-SMA contrary to YFO L-NAME rats. Other studies [25, 26] have described an increase in *α*-SMA expression in the aging heart, thus increasing the possibility that a transmitted paternal aging-related mechanism is behind the activation of fibroblasts and their transformation into myofibroblasts, under circulatory stress.

No significant increase in PDGFR*α*^+^ expression was seen in the young old father offspring hearts (OFO vs. OFO L-NAME). Since PDGFR*α*^+^ is selectively expressed in the adult hearts [27] with a central function in regulating the differentiation of epicardial-derived fibroblasts [28, 29], it seems that cardiac resident PDGFR*α*^+^ fibroblasts are not involved in aging-related fibrotic processes. Nonetheless, epicardial progenitor transcription factors, Tcf21, Tbx18, and Wt1, were all increased in OFO groups particularly in OFO L-NAME rats, similar to OF, confirming the epicardial origin of fibroblasts. These results are in accordance with the literature demonstrating that age-associated cardiac fibrosis is linked to dysregulation of resident mesenchymal fibroblasts [30], as well as recruitment and proliferation of epicardial fibroblasts in the myocardium [31].

TGF-*β* signaling pathway is one of the main pathways involved in cardiac fibrosis and its involvement has been widely described in the literature [32]. Western blot analysis of the hearts showed an increase in TGF-*β* expression in OFO L-NAME and OF rats, but not in YFO L-NAME rats, suggesting that paternal age increased the cardiac susceptibility to fibrosis following L-NAME treatment. In addition, only ERK1/2 was activated in OFO L-NAME. ERK1/2, a type of serine/threonine protein kinase in the downstream pathway of TGF-*β*, has vital involvement in the mitogen-activated protein kinase (MAPK) signaling pathway and has been shown to present cardiac phosphorylation levels proportional to the age of the animals [33]. Further analysis of the TFG-*β* signaling pathway using a phospho-protein array enabled a wider and more exhaustive analysis of the cardiac TGF-*β* pathway and led to the identification of an intricate protein-protein interaction network relating to the paternal age. TGF-*β* isoform 2 was identified as a protein cluster selectively activating TGFBR2 in aged hearts as well as in the OFO cardiac tissue treated with L-NAME. Downstream signaling included noncanonical TGF-*β* pathways, such as Pi3k, along with the Rac1-Pak1/2 pathways. TGFBR2 stimulated PKC and its downstream effector, ERK1/2. Finally, the tyrosine kinase Abl1 showed substantial paternal age dependency. TGF-*β* is involved in cellular senescence, but this signaling is largely dependent on cell type and cellular context [34]. Nonetheless, not only is there little information on the involvement of TGF-*β* in age-related cardiac fibrosis, but this is the first research to show intergenerational pivotal activation of TGF-*β*/MAPK noncanonical signaling pathways in the hearts of old father offspring.

Of the various epigenetic modifications, DNA methylation is the most well-known and studied process [35]. OFO rats showed a significant increase in overall cardiac DNA methylation and hydroxymethylation, thus confirming the presence of epigenetic modifications in the offspring's hearts. Further studies are needed to explore the role of DNA methylation in the pathogenesis of age-related fibrotic phenotype in the offspring's hearts.

## 5. Limitations

Our study presents some limitations. First, a follow-up and survival assessment was not carried out to evaluate the long-term effect of paternal age on the offspring. Other target organs of circulatory stress such as the kidneys could have been also examined. Second, our results lack genetic sequencing and evaluation of methylated genes related to the development of cardiac fibrosis. Finally, since TGF-*β* is known to be activated by oxidative stress and contributes to cellular aging and senescence [36], oxidative stress assessment in the blood and/or the heart tissue could be behind the activation of the TGF-*β* receptor pathway in cardiac fibrosis in our model.

## 6. Conclusions

This is the first study to identify the role advanced paternal age is in myocardial fibrosis by activation of the TGF-*β* signaling pathway in the offspring, under circulatory stress. An intergenerational transmission seems to be behind the paternal age-related early development of cardiac remodeling in the young offspring. Clinically, in young hypertensive progeny, advanced paternal age at conception may be a risk factor for early progression towards heart failure. Therefore, cardiovascular monitoring of the descendants should be considered in the event of an elderly parent union with a young one.

## Data Availability

All data generated or analyzed during this study are included in the published article. Any remaining datasets are available from the corresponding author upon reasonable request.

## References

[1] Bonnans C., Chou J., Werb Z. (2014). Remodelling the extracellular matrix in development and disease. *Nature Reviews. Molecular Cell Biology*.

[2] Khan S. S., Singer B. D., Vaughan D. E. (2017). Molecular and physiological manifestations and measurement of aging in humans. *Aging Cell*.

[3] Kong P., Christia P., Frangogiannis N. G. (2014). The pathogenesis of cardiac fibrosis. *Cellular and Molecular Life Sciences*.

[4] Meng X.-M., Nikolic-Paterson D. J., Lan H. Y. (2016). TGF-*β*: the master regulator of fibrosis. *Nature Reviews. Nephrology*.

[5] Zeisberg E. M., Tarnavski O., Zeisberg M. (2007). Endothelial-to-mesenchymal transition contributes to cardiac fibrosis. *Nature Medicine*.

[6] North B. J., Sinclair D. A. (2012). The intersection between aging and cardiovascular disease. *Circulation Research*.

[7] Frangogiannis N. G. (2019). Cardiac fibrosis: cell biological mechanisms, molecular pathways and therapeutic opportunities. *Molecular Aspects of Medicine*.

[8] Zhang W., Song M., Qu J., Liu G.-H. (2018). Epigenetic modifications in cardiovascular aging and diseases. *Circulation Research*.

[9] Grimaldi V., De Pascale M. R., Zullo A. (2017). Evidence of epigenetic tags in cardiac fibrosis. *Journal of Cardiology*.

[10] Stoll S., Wang C., Qiu H. (2018). DNA methylation and histone modification in hypertension. *International Journal of Molecular Sciences*.

[11] Grover M. M., Jenkins T. G. (2020). Transgenerational epigenetics: a window into paternal health influences on offspring. *The Urologic Clinics of North America*.

[12] Perez M. F., Lehner B. (2019). Intergenerational and transgenerational epigenetic inheritance in animals. *Nature Cell Biology*.

[13] Xie K., Ryan D. P., Pearson B. L. (2018). Epigenetic alterations in longevity regulators, reduced life span, and exacerbated aging-related pathology in old father offspring mice. *Proceedings of the National Academy of Sciences of the United States of America*.

[14] Carslake D., Tynelius P., van den Berg G. J., Davey Smith G. (2019). Associations of parental age with offspring all-cause and cause-specific adult mortality. *Scientific Reports*.

[15] Lee D. S., Pencina M. J., Benjamin E. J. (2006). Association of parental heart failure with risk of heart failure in offspring. *The New England Journal of Medicine*.

[16] Vik K. L., Romundstad P., Carslake D., Davey Smith G., Nilsen T. I. (2016). Transgenerational effects of parental cardiovascular disease and risk factors on offspring mortality: family-linkage data from the HUNT study, Norway. *European Journal of Preventive Cardiology*.

[17] Kazakov A., Hall R., Jagoda P. (2013). Inhibition of endothelial nitric oxide synthase induces and enhances myocardial fibrosis. *Cardiovascular Research*.

[18] Schiattarella G. G., Altamirano F., Tong D. (2019). Nitrosative stress drives heart failure with preserved ejection fraction. *Nature*.

[19] Szklarczyk D., Gable A. L., Lyon D. (2019). STRING v11: protein-protein association networks with increased coverage, supporting functional discovery in genome-wide experimental datasets. *Nucleic Acids Research*.

[20] Bartunek J., Weinberg E. O., Tajima M. (2000). Chronic N(G)-nitro-L-arginine methyl ester-induced hypertension: novel molecular adaptation to systolic load in absence of hypertrophy. *Circulation*.

[21] Ago T., Kuroda J., Pain J., Fu C., Li H., Sadoshima J. (2010). Upregulation of Nox4 by hypertrophic stimuli promotes apoptosis and mitochondrial dysfunction in cardiac myocytes. *Circulation Research*.

[22] Neilan T. G., Coelho-Filho O. R., Shah R. V. (2013). Myocardial extracellular volume fraction from T1 measurements in healthy volunteers and mice: relationship to aging and cardiac dimensions. *JACC: Cardiovascular Imaging*.

[23] Frangogiannis N. G. (2021). Cardiac fibrosis. *Cardiovascular Research*.

[24] Tarbit E., Singh I., Peart J. N., Rose’Meyer R. B. (2019). Biomarkers for the identification of cardiac fibroblast and myofibroblast cells. *Heart Failure Reviews*.

[25] Angelini A., Trial J., Ortiz-Urbina J., Cieslik K. A. (2020). Mechanosensing dysregulation in the fibroblast: a hallmark of the aging heart. *Ageing Research Reviews*.

[26] Kern S., Feng H.-Z., Wei H., Cala S., Jin J.-P. (2014). Up-regulation of alpha-smooth muscle actin in cardiomyocytes from non-hypertrophic and non-failing transgenic mouse hearts expressing N-terminal truncated cardiac troponin I. *FEBS Open Bio*.

[27] Ivey M. J., Kuwabara J. T., Riggsbee K. L., Tallquist M. D. (2019). Platelet-derived growth factor receptor-*α* is essential for cardiac fibroblast survival. *Am J Physiol-Heart Circ Physiol*.

[28] Rudat C., Norden J., Taketo M. M., Kispert A. (2013). Epicardial function of canonical Wnt-, Hedgehog-, Fgfr1/2-, and Pdgfra-signalling. *Cardiovascular Research*.

[29] Smith C. L., Baek S. T., Sung C. Y., Tallquist M. D. (2011). Epicardial-derived cell epithelial-to-mesenchymal transition and fate specification require PDGF receptor signaling. *Circulation Research*.

[30] Trial J., Heredia C. P., Taffet G. E., Entman M. L., Cieslik K. A. (2017). Dissecting the role of myeloid and mesenchymal fibroblasts in age-dependent cardiac fibrosis. *Basic Research in Cardiology*.

[31] Fang M., Xiang F.-L., Braitsch C. M., Yutzey K. E. (2016). Epicardium-derived fibroblasts in heart development and disease. *Journal of Molecular and Cellular Cardiology*.

[32] Frangogiannis N. G. (2022). Transforming growth factor-*β* in myocardial disease. *Nature Reviews. Cardiology*.

[33] Sunil K., Kakarla N. D. M. (2014). Molecular mechanisms of age-related cardiac hypertrophy in the F344XBN rat model. *Journal of Clinical & Experimental Cardiology*.

[34] Tominaga K., Suzuki H. I. (2019). TGF-*β* signaling in cellular senescence and aging-related pathology. *International Journal of Molecular Sciences*.

[35] Lev Maor G., Yearim A., Ast G. (2015). The alternative role of DNA methylation in splicing regulation. *Trends in Genetics*.

[36] Lyu G., Guan Y., Zhang C. (2018). TGF-*β* signaling alters H4K20me3 status via miR-29 and contributes to cellular senescence and cardiac aging. *Nature Communications*.

